# Complete Genome Sequence of *Corynebacterium pseudotuberculosis* Viscerotropic Strain N1

**DOI:** 10.1128/genomeA.01673-15

**Published:** 2016-01-28

**Authors:** Dan Loureiro, Ricardo W. Portela, Thiago J. Sousa, Flávia Rocha, Felipe L. Pereira, Fernanda A. Dorella, Alex F. Carvalho, Nildo Menezes, Eduardo S. Macedo, Lilia F. Moura-Costa, Roberto Meyer, Carlos A. G. Leal, Henrique C. Figueiredo, Vasco Azevedo

**Affiliations:** a Laboratory of Immunology and Molecular Biology, Federal University of Bahia, Salvador, Bahia, Brazil; b Laboratory of Cellular and Molecular Genetics, Federal University of Minas Gerais, Belo Horizonte, Minas Gerais, Brazil; c Aquacen, National Reference Laboratory for Aquatic Animal Diseases of Ministry of Fisheries and Aquaculture, Veterinary School, Federal University of Minas Gerais, Belo Horizonte, Minas Gerais, Brazil; d ARG Ltda., Equatorial Guinea, Oyala, Wele Nzas, Equatorial Guinea

## Abstract

We present the complete genome sequence of *Corynebacterium pseudotuberculosis* strain N1. The sequencing was performed with the Ion Torrent Personal Genome Machine system. The genome is a circular chromosome with 2,337,845 bp, a G+C content of 52.85%, and a total of 2,045 coding sequences, 12 rRNAs, 49 tRNAs, and 58 pseudogenes.

## GENOME ANNOUNCEMENT

*Corynebacterium pseudotuberculosis* is a Gram-positive bacterium that causes a chronic disease characterized by granuloma formation in subcutaneous and internal lymph nodes in goats and sheep (1). This disease is associated with direct economic losses and presents significant zoonotic potential (2). With the advent of next-generation sequencing platforms, new strains have been sequenced, and 22 strains are currently deposited in the database at the National Center for Biotechnology Information (NCBI). Increasing the number of deposited strains is fundamental to closing the pan-genome for this species and will aid in the development of new vaccines and treatment methods (3).

In this study, we present the complete genome sequence of *C. pseudotuberculosis* viscerotropic strain N1. The N1 strain was isolated from caseous material in the lung from a sheep that died in 2014 of apparent respiratory disease in Mongomo, Equatorial Guinea. The animal had no visible skin lesions and no veterinary medical history or presentation indicative of lymph node infarction. During the autopsy, all lymph nodes and organs were examined, and caseous lesions were identified only in the lungs. Furthermore, the sheep herd was observed for a period of 1.5 years and did show any lesions in the superficial lymph nodes; this would indicate that the isolated strain is viscerotropic. The biochemical test results for nitrate reductase were negative and therefore suggestive that this strain belongs to *C. pseudotuberculosis* biovar *ovis*. The genome was sequenced using the Ion Torrent Personal Genome Machine (PGM) system, a 400-bp fragment library kit, and a coverage of ~268.07-fold. The quality of the reads was analyzed using the FastQC software (http://www.bioinformatics.babraham.ac.uk/projects/fastqc), and *de novo* assembly was performed using SPAdes 3.6.0 (http://spades.bioinf.spbau.ru).

The assembly process produced 6 contigs and an *N*_50_ value of 543,043 bp. Scaffolding was performed with CONTIGuator 2.7 (4) using the genome of *C. pseudotuberculosis* strain 29156 (CP010795.1) as reference, and the gap-filling process was completed using SIMBA (http://ufmg-simba.sourceforge.net) and CLC Genomics Workbench 7.0 (Qiagen, USA) software. Automatic annotation was performed by transferring information from a curated database using in-house scripts. tRNAs, rRNAs, and some coding sequences (CDSs) absent in the transference by in-house scripts were predicted using RAST (http://rast.nmpdr.org/). All CDSs were manually curated using the Artemis software (5) and the UniProt database (http://www.uniprot.org).

The complete genome of *C. pseudotuberculosis* N1 includes one circular chromosome with a length of 2,337,845 bp, a G+C content of 52.85%, and a total of 2,045 CDSs, 12 rRNAs (5S, 16S, and 23S), 49 tRNAs, and 58 pseudogenes.

### Nucleotide sequence accession number.

The complete genome has been deposited in GenBank under the accession number CP013146.
